# Benefits of resistance exercise in lean women with fibromyalgia: involvement of IGF-1 and leptin

**DOI:** 10.1186/s12891-017-1477-5

**Published:** 2017-03-14

**Authors:** Jan L. Bjersing, Anette Larsson, Annie Palstam, Malin Ernberg, Indre Bileviciute-Ljungar, Monika Löfgren, Björn Gerdle, Eva Kosek, Kaisa Mannerkorpi

**Affiliations:** 1 Department of Rheumatology and Inflammation Research, Institute of Medicine, Sahlgrenska Academy, University of Gothenburg, Guldhedsgatan 10, Box 480, 40530 Gothenburg, Sweden; 2 Sahlgrenska University Hospital, Rheumatology, Gothenburg, Sweden; 3 University of Gothenburg Centre for Person Centered Care (GPCC), Gothenburg, Sweden; 4 Institute of Neuroscience and Physiology/Physiotherapy, Section of Clinical Neuroscience and Rehabilitation, Sahlgrenska Academy, University of Gothenburg, Gothenburg, Sweden; 5 Department of Dental Medicine and Scandinavian Center for Orofacial Neurosciences (SCON) Karolinska Institutet, Stockholm, Sweden; 6 Department of Clinical Sciences, Danderyd Hospital, Karolinska Institutet, Stockholm, Sweden; 7 Department of Medical and Health Sciences, Faculty of Medicine and Health Sciences, Linköping University, Pain and Rehabilitation Center, Anaesthetics, Operations and Specialty Surgery Center, Region Östergotland, Linköping, Sweden; 8 Department of Clinical Neuroscience, Karolinska Institutet and Stockholm Spine Center, Stockholm, Sweden; 9 Sahlgrenska University Hospital, Physiotherapy and Occupational therapy, Gothenburg, Sweden

## Abstract

**Background:**

Chronic pain and fatigue improves by exercise in fibromyalgia (FM) but underlying mechanisms are not known. Obesity is increased among FM patients and associates with higher levels of pain. Symptom improvement after aerobic exercise is affected by body mass index (BMI) in FM. Metabolic factors such as insulin-like growth factor 1 (IGF-1) and leptin may be involved. In this study, the aim was to evaluate the role of metabolic factors in lean, overweight and obese women during resistance exercise, in relation to symptom severity and muscle strength in women with FM.

**Methods:**

Forty-three women participated in supervised progressive resistance exercise, twice weekly for 15-weeks. Serum free and total IGF-1, IGF-binding protein 3 (IGFBP3), adiponectin, leptin and resistin were determined at baseline and after 15-weeks. Level of current pain was rated on a visual analogue scale (0–100 mm). Level of fatigue was rated by multidimensional fatigue inventory (MFI-20) subscale general fatigue (MFIGF). Knee extension force, elbow flexion force and handgrip force were assessed by dynamometers.

**Results:**

Free IGF-1 (*p* = 0.047), IGFBP3 (*p* = 0.025) and leptin (*p* = 0.008) were significantly decreased in lean women (*n* = 18), but not in the overweight (*n* = 17) and the obese (*n* = 8). Lean women with FM benefited from resistance exercise with improvements in current pain (*p*= 0.039, *n* = 18), general fatigue (MFIGF, *p* = 0.022, *n* = 18) and improved elbow-flexion force (*p* = 0.017, *n* = 18). In overweight and obese women with FM there was no significant improvement in pain or fatigue but an improvement in elbow flexion (*p* = 0.049; *p* = 0.012) after 15 weeks of resistance exercise.

**Conclusion:**

The clearest clinical response to resistance exercise was found in lean patients with FM. In these individuals, individualized resistance exercise was followed by changes in IGF-1 and leptin, reduced pain, fatigue and improved muscular strength. In overweight and obese women FM markers of metabolic signaling and clinical symptoms were unchanged, but strength was improved in the upper limb. Resistance exercise combined with dietary interventions might benefit patients with FM and overweight.

**Trial registration:**

The trial was registered 21 of October 2010 with ClinicalTrials.gov identification number: NCT01226784.

## Background

Fibromyalgia (FM) [1] is characterized by chronic pain, tenderness [2], and pain amplification [3–5]. Increased levels of inflammatory cytokines [6] and changes in neurotropic growth factors in the central nervous system and peripherally may influence the development and maintenance of central pain hypersensitivity by affecting adaptation and neuroplasticity [7–10]. This condition leads to considerable activity limitations and is very difficult to treat effectively.

Clinical experience and current research indicate that exercise is beneficial in FM and exercise was recently recommended as first line treatment ahead of pharmacological treatment [11]. However, meta-analysis in a Cochrane review of resistance exercise is based on few trials [12]. Planning exercise for patients with FM is challenging due to activity-induced pain at the initial phase both during isometric [13] and aerobic exercise [14]. However, we have previously reported positive changes in symptoms and strength after resistance exercise for the complete set of 130 patients participating in a multicenter randomized controlled trial [15]. Pain and strength [15] and fatigue were improved [16]. Furthermore, a number of independent studies indicate that resistance exercise for patients with FM is safe and effective [15, 17, 18].

Our previous studies show that improvement in symptoms after aerobic exercise was reduced and delayed among obese FM patients with apparent involvement of the metabolic factors, insulin-like growth factor (IGF-1) and leptin [7, 19]. However, a previous resistance exercise study showed unaltered levels of basal serum hormones including IGF-1 [20]. Obesity is common in FM, with a prevalence between 40 and 70% [21–23] and is correlated with higher levels of pain and fatigue [22, 24–26]. In the related syndrome of chronic fatigue, symptom severity is also associated with increased BMI and with the presence of metabolic syndrome [27]. There is an inverse relation between BMI and total IGF-1 levels [28, 29] and a deregulation of growth hormone/IGF-1 signaling in obesity [30, 31]. IGF-1 plays a key role in the adaptation to exercise [32] by regulating metabolic activity and cell proliferation in skeletal muscle and other peripheral tissues and in the central nervous system (CNS) [33, 34]. Up to one third of FM patients are estimated to suffer from growth hormone [35] deficiency and reduced IGF-1 [36–38].

Leptin is another important metabolic factor, it is a central regulator of satiety and body weight [39, 40] and is also involved in regulation of emotional responses [41–45] and pain [46]*.* Serum leptin is taken up into the CNS via the blood–brain barrier and the diurnal rhythm of leptin secretion is dependent on energy availability and is influenced by growth hormone, insulin and cortisol [47]. Leptin receptors are distributed in multiple regions in the CNS including the hippocampus, the hypothalamus [48] and multiple thalamic nuclei [49], reflecting the multiple roles of leptin.

The purpose of the study was to investigate how metabolic factors contribute to the effects of resistance exercise in patients with FM. Our hypothesis was that there may be a reduced response to resistance exercise in the overweight and obese women with FM compared to lean women and that the metabolic factors IGF-1 and leptin may be involved in this difference.

## Methods

### Study design

This is a substudy of a previously reported randomized controlled multicenter trial [15] (ClinicalTrials.gov identification number: NCT01226784) studying the effects of a progressive resistance exercise program [15]. This longitudinal and observatory substudy focused on biological and clinical changes after resistance exercise.

The rationale of the resistance exercise program was to improve muscle strength and health status by progressive resistance exercise, but to minimize the risk of increased pain while loading the muscles. The 15-week exercise program twice a week has previously been described in detail [15]. Exercise was performed under the supervision of experienced physiotherapists according to the principles of person-centered care [50]. Thus, the exercise program and its progression was individually planned with each patient and modified according to individual resources. Exercise was preceded by an individual meeting to discuss the patient’s goals, her previous experiences and possible obstacles for exercise. One repetition maximum (1RM) was tested, and the initial load of each exercise was defined with each patient and starting at 40% of 1RM. Each session was initiated with a 10 min warm-up period, followed by resistance exercise for legs, arms and hands and core stability and ended with stretching exercise. After 3–4 weeks the load was increased to 60% and thereafter to 80% of 1RM. Explosive strength exercises for legs were included at 5 and 8 weeks, as described previously [15]. Exercise was conducted in groups of 5–7 patients and lasted for about 1 hour.

### Participants

#### Criteria for inclusion

Women with FM, aged 20 to 65 years and who were able to participate in the assigned exercise twice a week for 15 weeks. The women were screened for eligibility by an experienced physician to verify ACR 1990 criteria for FM by means of a standardized interview and palpation of tender points [2]. Participation with blood samples was optional in the primary trial. All participants were offered to participate with blood samples and the ability to participate with blood samples at baseline and after the exercise period [15] was an additional inclusion criteria in this substudy.

#### Criteria for exclusion

As described previously [15], exclusion criteria were high blood pressure (>160/90 mmHg), osteoarthritis in hip or knee, confirmed by radiological findings and affecting activities of daily life such as stair climbing or walking, other severe somatic or psychiatric disorders, other dominating causes of pain than FM, high consumption of alcohol (alcohol use disorders identification test (AUDIT) score >6) [32], participation in a rehabilitation program within the past year, regular resistance exercise or relaxation exercise twice a week or more, inability to understand or speak Swedish, and not being able to refrain from analgesics, non-steroidal anti-inflammatory drugs (NSAIDs) or hypnotic drugs for 48 h prior to examinations.

Forty-three women with FM, were examined at baseline and after 15 weeks of the intervention (post-test). Serum was collected at rest at baseline and at post-test.

For patient characteristics, see Table 1. Lean patients were defined as BMI below 25 kg/m^2^ and BMI ranged from 20.9 to less than 25.0 kg/m^2^; overweight patients had BMI from 25.0 to 29.9 kg/m^2^. Obese patients had BMI **≥**30.0 kg/m^2^, with a range of 30.3 to 39.5 kg/m^2^ [51].

**Table 1:** Characteristics of the study population

Characteristics	All patients *n* = 43	Lean *n* = 18	Overweight *n* = 17	Obese *n* = 8	*p* Value^a^ Lean vs overweight	*p* Value^a^ Lean vs obese	*p* Value^a^ Overweight vs obese
Age (years)	51 (25 to 64)	50 (25 to 63)	53 (34 to 64)	51 (25 to 63)	0.351	0.807	0.711
BMI (kg/m^2^)	25.6 (20.9 to 39.9)	23.1 (20.9 to 24.96)	26.2 (25.1 to 29.9)	35.2 (30.8 to 39.9)	**<0.001**	**<0.001**	**<0.001**
Symptom duration (years)	9 (0 to 35)	8 (1 to 20)	10 (0 to 35)	7 (1 to 26)	0.386	0.892	0.628
Tender points (n)	16 (11 to 18)	16 (12 to 18)	16 (11 to 18)	17 (15 to 18)	0.909	0.311	0.238
Pharmacologic treatment, N (%)					*p* Value^b^ Lean vs overweight	*p* Value^b^ Lean vs obese	*p* Value^b^ Overweight vs obese
NSAID/paracetamol	34 (79)	12 (67)	15 (88)	7 (88)	0.264	0.531	0.958
Opioids for mild to moderate pain. Yes	6 (14)	2 (11)	3 (18)	1 (12)	0.945	0.918	0.743
Antidepressants. Yes	22 (51)	8 (44)	11 (65)	3 (38)	0.3880	0.741	0.397
Anticonvulsives. Yes	4 (9)	2 (11)	2 (6)	1 (12)	0.9516	0.918	0.958
Sedatives. Yes	7 (16)	3(17)	4 (24)	0 (0)	0.9326	0.574	0.362

Lean patients had BMI from 20.9 to < 25.0 kg/m^2^; overweight patients had BMI 25.0 to 29.9 kg/m^2^. Obese patients had BMI **≥**30.0 kg/m^2^. Median values and range (min, max). Furthest to the right is shown group comparisons (*p*-value; ^a^Mann-Whitney *U*-test. ^b^Chi-square test with Yates correction). *P*-value in bold type is significant

### Clinical measurements

Current pain at the time of interview was rated on a visual analogue scale (0–100). Fatigue during the previous week was rated with the Multidimensional Fatigue Inventory (MFI-20) [52] subscale of General Fatigue (MFIGF, range 4–20), which estimates fatigue by questions related to feeling “fit”, “tired” and “rested”. A higher score indicates more severe pain or fatigue.

Maximal isometric knee extension force (N) was measured with *Steve Strong® (Stig Starke HBI, Göteborg, Sweden)*, a dynamometer. The participant was in a fixed seated position with knee and hip in 90° of flexion. A non-elastic strap was attached to a pressure transducer with an amplifier. A mean value of three trials from the right and left leg was calculated [53, 54]. Average maximal isometric elbow flexion force (kg) was measured with *Isobex® (Medical Device Solutions AG, Oberburg, Switzerland)*. The upper arm was aligned with the trunk and the elbow in 90° of flexion [55]. A mean value from the right and left elbow flexion was calculated. *Grippit® (AB Detektor, Göteborg, Sweden)* is an electronic instrument that measures hand grip force (N). The mean force over 10 seconds was recorded [56].

### Laboratory analysis

Serum samples were acquired by venipuncture of the cubital vein. Collected blood samples were centrifuged at 1500 g for 30 min immediately after collection, aliquoted, and stored frozen at −70 °C until use. Biological markers were analyzed by sandwich enzyme-linked immunosorbent assays (ELISAs) using a pair of specific antibodies for human adiponectin (DY1065, 62 pg/mL), human leptin (DY389, 31 pg/mL), human resistin (DY1359, 10 pg/mL), human serum free bioactive IGF-1 (DY291, 4 pg/mL) and IGFBP3 (DY675, 0.125 ng/ml) which were all purchased from RnD Systems (Minneapolis, MN, USA). All assays were performed according to the instructions of the manufacturers. ELISAs were read with a Spectramax 340 from Molecular Devices (Sunnyvale, CA, USA). Serum total IGF-1 was measured by solid-phase, enzyme-labeled chemoluminescent immunoassay with IDS-iSYS IGF1 immunoassay (IS-3900, Immunodiagnostic Systems Boldon, UK) using the IDS-iSYS Multi-Discipline Immunoassay System (IS-310400, Immunodiagnostic Systems Boldon, UK).

### Statistics

Descriptive data are presented as median and interquartile range (IQR). ∆-values represent the value of change between baseline and post-treatment examination. The Wilcoxon signed-rank test was used for comparisons of continuous variables within groups. Comparisons between groups were made using Mann–Whitney *U*-test. Effect size (Cohen’s d) was calculated as d = (Mean after exercise-mean at baseline)/Pooled standard deviation. Chi-square test was used for comparison of categorical variables (pharmaceutical treatment). To control for possible type I errors, the upper limit of the expected number of false significant results for the analyses was calculated by the following formula:

$$ \left(\mathrm{Numberoftests}{\textstyle \hbox{-}}\mathrm{Numberofsignificanttests}\right)\times \alpha /\left(1{\textstyle \hbox{-}}\alpha \right), $$

where α is the significance level [57]. All significance tests were two-sided and conducted at the 5% significance level. All significant tests were two-tailed, and values of *p* < 0.05 were considered significant. All statistical evaluation of data was done with the statistic program IBM SPSS Statistics for Macintosh, Version 22.0 (IBM Corporation, Armonk, New York, USA).

## Results

The participant characteristics are presented in Table 1.

### IGF-1 and adipokines

Baseline levels and change in IGF-1 and adipokines are presented in Table 2. In the whole group, total IGF-1 (*p* = 0.018), IGFBP3 (*p* = 0.045) and leptin (*p* = 0.040) were reduced after 15 weeks. In parallel, free IGF-1 (*p* = 0.047), IGBP3 (*p* = 0.025) and leptin (*p* = 0.008) were significantly decreased in lean patients, but not in the overweight and the obese. Change in free IGF-1 was significantly different between lean and obese individuals after resistance exercise (*p* = 0.035). Change in leptin differed significantly between lean and overweight (*p* = 0.005). Changes in total-IGF-1 and IGFBP3 did not differ significantly between the groups.

**Table 2:** Serum levels of total IGF-1, serum free IGF-1, IGFBP3, adiponectin, leptin, and resistin at baseline, and change (∆) at posttest after resistance exercise

	All patients		Lean		Overweight		Obese		Intergroup differences in change		
	Baseline	∆Posttest	Baseline	∆Posttest	Baseline	∆Posttest	Baseline	∆Posttest	Lean vs overweight	Lean vs obese	Overweight vs obese
	Median (IQR)	Median (IQR)	Median (IQR)	Median (IQR)	Median (IQR)	Median (IQR)	Median (IQR)	Median (IQR)			
		Cohen’s d		Cohen’s d		Cohen’s d		Cohen’s d			
		*P*-value^a^		*P*-value^a^		*P*-value^a^		*P*-value^a^	*P*-value^b^	*P*-value^b^	*P*-value^b^
Total IGF-1	137 (54)	−3 (−21.2 to 5)	135 (54)	−11 (−18,8 to 5)	151 (61)	−3 (−47,5 to 7)	137 (61)	−1.5 (−18 to 8,8)			
(ng/ml)	*n* = 42	*n* = 42	*n* = 18	*n* = 18	*n* = 16	*n* = 16	*n* = 8	*n* = 8			
		−0.23		−0.2		−0.36		−0.09			
		**0.018**		*p* = 0.076		*p* = 0.147		*p* = 0.528	*p* = 0.986	*p* = 0.531	*p* = 0.528
Free IGF-1	2.6 (3.2)	0 (−1.4 to 1.0)	3.3 (2.3)	−0.7 (−1,9 to 0,1)	2.6 (3.3)	0.4 (−1,1 to 1,5)	0.8 (2.3)	0.4 (−0,6 to 2,8)			
(ng/ml)	*n* = 43	*n* = 43	*n* = 18	*n* = 18	*n* = 17	*n* = 17	*n* = 8	*n* = 8			
		−0.05		−0.32		0.1		0.6			
		0.752		***p*** **= 0.047**		*p* = 0.485		*p* = 0.237	*p* = 0.053	***p*** **= 0.035**	*p* = 0.511
IGFBP3	823 (159)	−46.4 (−84.4 to 33.2)	828 (86)	−56 (−97 to 5)	790 (161)	−47 (−86 to 119)	858 (238)	−41 (−73 to 23)			
(ng/ml)	*n* = 43	*n* = 43	*n* = 18	*n* = 18	*n* = 17	*n* = 17	*n* = 8	*n* = 8			
		−0.29		−0.57		0.02		−0.36			
		***p*** **= 0.045**		***p*** **= 0.025**		*p* = 0.943		*p* = 0.161	*p* = 0.335	*p* = 0.567	*p* = 0.887
Adiponectin	10288 (6280)	192 (−1880 to 2064)	12036 (7396)	224 (−1638 to 2406)	11624 (5668)	−832 (−1948 to 1752)	6600 (4020)	−204 (−2648 to 2398)			
(ng/ml)	*n* = 43	*n* = 43	*n* = 18	*n* = 18	*n* = 17	*n* = 17	*n* = 8	*n* = 8			
		0.02		0.09		−0.03		−0.04			
		0.819		*p* = 0.586		*p* = 0.813		*p* = 0.889	*p* = 0.59	*p* = 0.765	*p* = 0.887
Leptin	27.7 (71.6)	−4.9 (−18.5 to 1.0)	21 (86)	−15.9 (−23,6 to −0,1)	39 (60)	0 (−3 to 19,6)	23 (106)	−12 (−23,1 to −2,3)			
(ng/ml)	*n* = 43	*n* = 43	*n* = 18	*n* = 18	*n* = 17	*n* = 17	*n* = 8	*n* = 8			
		−0.13		−0.22		−0.02		−0.51			
		**0.040**		***p*** **= 0.008**		*p* = 0.463		*p* = 0.093	***p*** **= 0.005**	*p* = 0.849	***p*** **= 0.019**
Resistin	13.1 (5.1)	−0.7 (−2.0 to 0.9)	13 (5)	−0.7 (−1.5 to 1)	11 (7)	0.2 (−2,5 to 1,6)	15 (5)	−1.6 (−1,9 to 0,1)			
(ng/ml)	*n* = 43	*n* = 43	*n* = 18	*n* = 18	*n* = 17	*n* = 17	*n* = 8	*n* = 8			
		−0.11		0.04		−0.19		−0.42			
		0.227		*p* = 0.446		*p* = 0.723		*p* = 0.161	*p* = 0.987	*p* = 0.285	*p* = 0.588

Serum levels of the whole group and subdivided in lean, overweight and obese patients. Median values, upper and lower boundaries of the interquartile (IQR) range are indicated. Effect size of change is shown as Cohen’s d. Within group comparisons (*p*-value^a^: Wilcoxon signed rank test) and group comparisons (*p*-value^b^: Mann–Whitney *U*-test) are shown. *P*-values in bold type are significant

### Symptom severity and strength

Changes after resistance exercise in symptom severity and strength was assessed in lean (*n* = 18), overweight (*n* = 17) and obese individuals (*n* = 8), see Table 3. Following resistance exercise the lean patients with FM improved with regard to current pain (*p* = 0.039) and general fatigue (∆MFIGF, *p* = 0.022). Elbow flexion force was also significantly improved in this group (*p* = 0.017) as well as in overweight (*p* = 0.049) and obese patients (*p* = 0.012). Symptoms did not improve in the overweight and the obese women. Changes in symptoms and muscle strength did not differ significantly between the groups.

**Table 3:** Clinical response to resistance exercise among lean, overweight and obese patients

	Lean		Overweight		Obese		Differences in change		
	Baseline	∆Posttest	Baseline	∆Posttest	Baseline	∆Posttest	Lean vs overweight	Lean vs obese	Overweight vs obese
	Median (IQR)	Median (IQR)	Median (IQR)	Median (IQR)	Median (IQR)	Median (IQR)			
		*P*-value^a^		*P*-value^a^		*P*-value^a^	*P*-value^b^	*P*-value^b^	*P*-value^b^
Current pain	56 (43)	−14.5 (−25,3 to 2,8)	48 (39)	−13 (−31,5 to 13,5)	49 (17)	1 (−37,3 to 16,5)			
(VAS)	*n* = 18	*n* = 18	*n* = 17	*n* = 17	*n* = 8	*n* = 8			
		***p*** **= 0.039**		*p* = 0.136		*p* = 0.624	*p* = 0.987	*p* = 0.429	*p* = 0.549
MFIGF fatigue	19 (2)	−1 (−3 to 0)	18 (4)	−1 (−2 to 1)	19 (4)	−0.5 (−2,8 to 1)			
(4–20)	*n* = 18	*n* = 18	*n* = 17	*n* = 17	*n* = 8	*n* = 8			
		***p*** **= 0.022**		*p* = 0.303		*p* = 0.443	*p* = 0.757	*p* = 0.567	*p* = 0.842
Hand grip force	174 (110)	8.8 (−6,4 to 30,4)	165 (122)	10.3 (−7,5 to 41,3)	173 (144)	14 (1,4 to 35,1)			
(N)	*n* = 18	*n* = 18	*n* = 17	*n* = 16	*n* = 8	*n* = 8			
		*p* = 0.074		*p* = 0.098		*p* = 0.123	*p* = 0.851	*p* = 0.567	*p* = 0.881
Elbow flexion force	12.9 (5.1)	1.2 (0 to 3,6)	11.1 (7.1)	1.4 (−0,8 to 5,1)	13.5 (10.6)	3 (1,6 to 5,1)			
(kg)	*n* = 18	*n* = 18	*n* = 17	*n* = 17	*n* = 8	*n* = 8			
		***p*** **= 0.017**		***p*** **= 0.049**		***p*** **= 0.012**	*p* = 0.858	*p* = 0.080	*p* = 0.315
Knee extension force	338 (135)	11.5 (−46,6 to 48,3)	306 (136)	43 (−31 to 74,3)	389 (185)	35.3 (−16,3 to 105,6)			
(N)	*n* = 18	*n* = 18	*n* = 17	*n* = 17	*n* = 8	*n* = 8			
		*p* = 0.647		*p* = 0.113		*p* = 0.208	*p* = 0.303	*p* = 0.285	*p* = 0.754

Symptom severity and strength at baseline, and change (∆) at posttest after resistance exercise. Median values with upper and lower boundaries of the interquartile range are indicated Within group comparisons (*p*-value^a^: Wilcoxon signed rank test) and group comparisons (*p*-value^b^: Mann–Whitney *U*-test) are presented. *P*-values in bold type are significant

### Type 1 error

Changes in IGF-1 and adipokines resistance exercise in the whole group and among lean, overweight and obese patients (Table 2) comprised a total of 42 comparisons and the upper level of the number of false significant results was 1.7, which means that two of the nine significant results might be false.

Clinical response to resistance exercise among lean, overweight and obese patients (Table 3) comprised a total of 30 comparisons and the upper level of the number of false significant results was 1.3, which means that one of the five significant results might be false.

## Discussion

Recent publications have recommended resistance exercise for patients with FM [17, 18]. Since muscle strength is reduced in many women with FM, graded resistance exercise adjusted to health status and symptoms, appears to be important. Women with FM participating in a resistance exercise program have been found to improve in both symptoms and muscular strength [15]. In the present substudy, the levels of free IGF-1, IGFBP3 and leptin were reduced in lean women with FM after 15 weeks of exercise, along with improvement in pain, fatigue and upper limb muscle strength. In overweight and obese women, levels of IGF-1 and adipokines as well as pain and fatigue were unchanged while upper limb muscle strength was increased.

The improvement in lean women with FM found in the present study is in line with a previous study where fatigue was reduced in lean women with FM after 15 weeks of aerobic exercise while symptom improvements were delayed in overweight and obese women with FM [7]. In the same study, resting levels of IGFBP3 also tended to decrease in lean women with FM, free IGF-1 was unchanged while total IGF-1 increased following aerobic exercise [7]. Resistance exercise by pharmacologically androgen-deprived men led to reduced IGF-1 and IGFBP3 and normalized leptin and adiponectin levels [58] but IGF-1 levels were not altered in a study of resistance exercise in elderly women with FM [20].

Changes in the metabolic factors IGF-1 and leptin in response to exercise may affect pain processing in the CNS. Recent studies indicate the involvement of hippocampus in response to exercise and in chronic pain. In FM, impaired executive function associates with reduced hippocampal activation [59] and connectivity is decreased between pain areas and sensorimotor brain areas [60]. The hippocampus is involved in chronic pain and FM [61, 62], participates in pain processing [35, 63–66] and indicates neurotropic changes in FM [67, 68] and chronic pain [69, 70]. However, regular exercise leads to functional and neurotropic changes in the hippocampus [71, 72] and normalization of functional connectivity in women with FM [73]. Hippocampal neurogenesis and neural plasticity is modulated by IGF-1 and other metabolic signals [34]. The majority of studies on physical activity and the CNS involve aerobic exercise but resistance exercise has shown similar benefit in the CNS in terms of cognitive function [74] and changes in the growth factors IGF-1 and brain-derived neurotrophic factor [75]. Furthermore, in a group of elderly individuals physical activity levels but not aerobic fitness correlated with cognitive performance, increased prefrontal and cingulate gray matter and with levels of neurotrophic factor G-CSF [76]. This indicates that resistance exercise will be sufficient and that cardiovascular exercise is not required. In concurrence, the beneficial effects of both resistance and aerobic exercise in FM on pain and fatigue may involve neurotropic and neuroprotective signaling in the hippocampus mediated by leptin [72] and adaptation to exercise-induced peaks of IGF-1 [19, 77–79].

High levels of leptin are suggested as a marker of leptin resistance involving both CNS and the periphery [80]. Acute aerobic exercise downregulated leptin transcription in adipose tissue [81] and leptin sensitivity in the CNS was improved [82]. Decreased leptin levels following exercise may therefore indicate increased leptin sensitivity. In agreement, reduced leptin levels after 3 months [83] and 6 months of resistance exercise was previously reported [84]. Thus, improved leptin signaling seems to associate with exercise and reduced pain and fatigue.

Obesity is associated with reduced leptin sensitivity [85]. In patients with type 2 diabetes, leptin levels were not altered after 6 weeks of resistance exercise [86]. Possibly three months of progressively increased resistance exercise is also too short a duration to improve leptin receptor sensitivity in overweight patients. Thus, a longer period of exercise up to 6 months may be beneficial in patients with obesity.

The main limitation of this study is the small sample size of the BMI groups. However, the present results indicate that IGF-1 and leptin are involved in change of pain and fatigue in patients with FM after resistance exercise.

## Conclusions

The clearest clinical response to resistance exercise was found in lean women with FM. In these individuals, individualized resistance exercise was followed by changes in IGF-1 and leptin, reduced pain, fatigue and improved upper limb muscular strength. In overweight and obese women with FM, markers of metabolic signaling and clinical symptoms were unchanged, but strength was improved. Resistance exercise combined with dietary interventions might benefit patients with FM and overweight.

## Abbreviations

1RM: One repetition maximum
BMI: Body mass index
CNS: Central nervous system
FM: Fibromyalgia
IGF-1: Insulin-like growth factor 1
IGFBP3: IGF-binding protein 3
MFIGF: Multidimensional fatigue inventory (MFI-20) subscale general fatigue
NSAIDs: Non-steroidal anti-inflammatory drugs

## References

[1] Michiels VCR, Fischler B, Hoffmann G, Le Bon O (1996). Cognitive functioning in patients with chronic fatigue syndrome. J Clin Exp Neuropsychol.

[2] Wolfe F, Smythe HA, Yunus MB, Bennett RM, Bombardier C, Goldenberg DL, Tugwell P, Campbell SM, Abeles M, Clark P (1990). The American College of Rheumatology 1990 Criteria for the Classification of Fibromyalgia. Report of the Multicenter Criteria Committee. Arthritis Rheum.

[3] Kosek E, Ekholm J, Hansson P (1996). Sensory dysfunction in fibromyalgia patients with implications for pathogenic mechanisms. Pain.

[4] Lannersten L, Kosek E (2010). Dysfunction of endogenous pain inhibition during exercise with painful muscles in patients with shoulder myalgia and fibromyalgia. Pain.

[5] Kosek E, Ekholm J, Hansson P (1996). Modulation of pressure pain thresholds during and following isometric contraction in patients with fibromyalgia and in healthy controls. Pain.

[6] Kadetoff D, Lampa J, Westman M, Andersson M, Kosek E (2012). Evidence of central inflammation in fibromyalgia-increased cerebrospinal fluid interleukin-8 levels. J Neuroimmunol.

[7] Bjersing JL, Erlandsson M, Bokarewa MI, Mannerkorpi K (2013). Exercise and obesity in fibromyalgia. Beneficial roles of insulin-like growth factor 1 and resistin?. Arthritis Res Ther.

[8] Garcia-Segura LM, Diz-Chaves Y, Perez-Martin M, Darnaudery M (2007). Estradiol, insulin-like growth factor-I and brain aging. Psychoneuroendocrinology.

[9] Morgado C, Silva L, Pereira-Terra P, Tavares I (2011). Changes in serotoninergic and noradrenergic descending pain pathways during painful diabetic neuropathy: the preventive action of IGF1. Neurobiol Dis.

[10] Fasick V, Spengler RN, Samankan S, Nader ND, Ignatowski TA (2015). The hippocampus and TNF: Common links between chronic pain and depression. Neurosci Biobehav Rev.

[11] Macfarlane GJ, Kronisch C, Dean LE, Atzeni F, Hauser W, Fluss E, Choy E, Kosek E, Amris K, Branco J (2017). EULAR revised recommendations for the management of fibromyalgia. Ann Rheum Dis.

[12] Busch AJ, Webber SC, Richards RS, Bidonde J, Schachter CL, Schafer LA, Danyliw A, Sawant A, Dal Bello-Haas V, Rader T (2013). Resistance exercise training for fibromyalgia. Cochrane Database Syst Rev.

[13] Staud R, Robinson ME, Price DD (2005). Isometric exercise has opposite effects on central pain mechanisms in fibromyalgia patients compared to normal controls. Pain.

[14] Mannerkorpi K, Nordeman L, Cider A, Jonsson G (2010). Does moderate-to-high intensity Nordic walking improve functional capacity and pain in fibromyalgia? A prospective randomized controlled trial. Arthritis Res Ther.

[15] Larsson A, Palstam A, Löfgren M, Ernberg M, Bjersing J, Bileviciute-Ljungar I, Gerdle B, Kosek E, Mannerkorpi K (2015). Resistance exercise improves muscle strength, health status and pain intensity in fibromyalgia—a randomized controlled trial. Arthritis Res Ther.

[16] Ericsson A, Palstam A, Larsson A, Lofgren M, Bileviciute-Ljungar I, Bjersing J, Gerdle B, Kosek E, Mannerkorpi K (2016). Resistance exercise improves physical fatigue in women with fibromyalgia: a randomized controlled trial. Arthritis Res Ther.

[17] Jones KD (2015). Recommendations for resistance training in patients with fibromyalgia. Arthritis Res Ther.

[18] Nelson NL (2015). Muscle strengthening activities and fibromyalgia: a review of pain and strength outcomes. J Bodyw Mov Ther.

[19] Bjersing JL, Dehlin M, Erlandsson M, Bokarewa MI, Mannerkorpi K (2012). Changes in pain and insulin-like growth factor 1 in fibromyalgia during exercise: the involvement of cerebrospinal inflammatory factors and neuropeptides. Arthritis Res Ther.

[20] Valkeinen H, Hakkinen K, Pakarinen A, Hannonen P, Hakkinen A, Airaksinen O, Niemitukia L, Kraemer WJ, Alen M (2005). Muscle hypertrophy, strength development, and serum hormones during strength training in elderly women with fibromyalgia. Scand J Rheumatol.

[21] Bennett RM, Jones J, Turk DC, Russell IJ, Matallana L (2007). An internet survey of 2,596 people with fibromyalgia. BMC Musculoskelet Disord.

[22] Neumann L, Lerner E, Glazer Y, Bolotin A, Shefer A, Buskila D (2008). A cross-sectional study of the relationship between body mass index and clinical characteristics, tenderness measures, quality of life, and physical functioning in fibromyalgia patients. Clin Rheumatol.

[23] Okifuji A, Bradshaw DH, Olson C (2009). Evaluating obesity in fibromyalgia: neuroendocrine biomarkers, symptoms, and functions. Clin Rheumatol.

[24] Kim CH, Luedtke CA, Vincent A, Thompson JM, Oh TH (2012). Association of body mass index with symptom severity and quality of life in patients with fibromyalgia. Arthritis Care Res.

[25] Mork PJ, Vasseljen O, Nilsen TI (2010). Association between physical exercise, body mass index, and risk of fibromyalgia: longitudinal data from the Norwegian Nord-Trondelag Health Study. Arthritis Care Res.

[26] Okifuji A, Donaldson GW, Barck L, Fine PG (2010). Relationship between fibromyalgia and obesity in pain, function, mood, and sleep. J Pain.

[27] Maloney EM, Boneva RS, Lin JM, Reeves WC (2010). Chronic fatigue syndrome is associated with metabolic syndrome: results from a case–control study in Georgia. Metab Clin Exp.

[28] Faupel-Badger JM, Berrigan D, Ballard-Barbash R, Potischman N (2009). Anthropometric correlates of insulin-like growth factor 1 (IGF-1) and IGF binding protein-3 (IGFBP-3) levels by race/ethnicity and gender. Ann Epidemiol.

[29] Friedrich N, Jorgensen T, Juul A, Spielhagen C, Nauck M, Wallaschofski H, Linneberg A (2012). Insulin-like growth factor I and anthropometric parameters in a Danish population. Exp Clin Endocrinol Diabetes.

[30] Vahl N, Jorgensen JO, Skjaerbaek C, Veldhuis JD, Orskov H, Christiansen JS (1997). Abdominal adiposity rather than age and sex predicts mass and regularity of GH secretion in healthy adults. Am J Phys.

[31] Pijl H, Langendonk JG, Burggraaf J, Frolich M, Cohen AF, Veldhuis JD, Meinders AE (2001). Altered neuroregulation of GH secretion in viscerally obese premenopausal women. J Clin Endocrinol Metab.

[32] Adams GR (2002). Invited Review: Autocrine/paracrine IGF-I and skeletal muscle adaptation. J Appl Physiol.

[33] Rafalski VA, Brunet A (2011). Energy metabolism in adult neural stem cell fate. Prog Neurobiol.

[34] Mainardi M, Fusco S, Grassi C (2015). Modulation of hippocampal neural plasticity by glucose-related signaling. Neural Plast.

[35] Covey WC, Ignatowski TA, Knight PR, Spengler RN (2000). Brain-derived TNFalpha: involvement in neuroplastic changes implicated in the conscious perception of persistent pain. Brain Res.

[36] Bennett RM, Cook DM, Clark SR, Burckhardt CS, Campbell SM (1997). Hypothalamic-pituitary-insulin-like growth factor-I axis dysfunction in patients with fibromyalgia. J Rheumatol.

[37] Cuatrecasas G, Gonzalez MJ, Alegre C, Sesmilo G, Fernandez-Sola J, Casanueva FF, Garcia-Fructuoso F, Poca-Dias V, Izquierdo JP, Puig-Domingo M (2010). High prevalence of growth hormone deficiency in severe fibromyalgia syndromes. J Clin Endocrinol Metab.

[38] Bennett RM (2002). Adult growth hormone deficiency in patients with fibromyalgia. Curr Rheumatol Rep.

[39] Zhang Y, Proenca R, Maffei M, Barone M, Leopold L, Friedman JM (1994). Positional cloning of the mouse obese gene and its human homologue. Nature.

[40] Kamohara S, Burcelin R, Halaas JL, Friedman JM, Charron MJ (1997). Acute stimulation of glucose metabolism in mice by leptin treatment. Nature.

[41] Chehab FF (2000). Leptin as a regulator of adipose mass and reproduction. Trends Pharmacol Sci.

[42] Lu XY, Kim CS, Frazer A, Zhang W (2006). Leptin: a potential novel antidepressant. Proc Natl Acad Sci U S A.

[43] Liu J, Garza JC, Bronner J, Kim CS, Zhang W, Lu XY (2010). Acute administration of leptin produces anxiolytic-like effects: a comparison with fluoxetine. Psychopharmacology.

[44] Haque Z, Akbar N, Yasmin F, Haleem MA, Haleem DJ (2013). Inhibition of immobilization stress-induced anorexia, behavioral deficits, and plasma corticosterone secretion by injected leptin in rats. Stress.

[45] Guo M, Huang TY, Garza JC, Chua SC, Lu XY (2013). Selective deletion of leptin receptors in adult hippocampus induces depression-related behaviours. Int J Neuropsychopharmacol.

[46] Maeda T, Kiguchi N, Kobayashi Y, Ikuta T, Ozaki M, Kishioka S (2009). Leptin derived from adipocytes in injured peripheral nerves facilitates development of neuropathic pain via macrophage stimulation. Proc Natl Acad Sci U S A.

[47] Berggren JR, Hulver MW, Houmard JA (2005). Fat as an endocrine organ: influence of exercise. J Appl Physiol.

[48] Bonda DJ, Stone JG, Torres SL, Siedlak SL, Perry G, Kryscio R, Jicha G, Casadesus G, Smith MA, Zhu XW (2014). Dysregulation of leptin signaling in Alzheimer disease: evidence for neuronal leptin resistance. J Neurochem.

[49] Elmquist JK, Bjorbaek C, Ahima RS, Flier JS, Saper CB (1998). Distributions of leptin receptor mRNA isoforms in the rat brain. J Comp Neurol.

[50] Ekman I, Swedberg K, Taft C, Lindseth A, Norberg A, Brink E, Carlsson J, Dahlin-Ivanoff S, Johansson IL, Kjellgren K (2011). Person-centered care--ready for prime time. Eur J Cardiovasc Nurs.

[51] National Cholesterol Education Program (NCEP) Coordinating Committee. Clinical Guidelines on the Identification, Evaluation, and Treatment of Overweight and Obesity in Adults--The Evidence Report. National Institutes of Health. Obes Res. 1998;6(Suppl 2):51S-209S. 9813653

[52] Ericsson A, Mannerkorpi K (2007). Assessment of fatigue in patients with fibromyalgia and chronic widespread pain. Reliability and validity of the Swedish version of the MFI-20. Disabil Rehabil.

[53] Brodin E, Ljungman S, Sunnerhagen KS (2008). Rising from a chair: a simple screening test for physical function in predialysis patients. Scand J Urol Nephrol.

[54] Schaufelberger M, Eriksson B, Grimby G, Held P, Swedberg K (1997). Skeletal muscle alterations in patients with chronic heart failure. Eur Heart J.

[55] Leggin BG, Neuman RM, Iannotti JP, Williams GR, Thompson EC (1996). Intrarater and interrater reliability of three isometric dynamometers in assessing shoulder strength. J Shoulder Elbow Surg.

[56] Nordenskiold UM, Grimby G (1993). Grip force in patients with rheumatoid arthritis and fibromyalgia and in healthy subjects. A study with the Grippit instrument. Scand J Rheumatol.

[57] Eklund G, Seeger P (1965). Massignifikansanalys. Statistisk tidskrift.

[58] Mina DS, Connor MK, Alibhai SM, Toren P, Guglietti C, Matthew AG, Trachtenberg J, Ritvo P (2013). Exercise effects on adipokines and the IGF axis in men with prostate cancer treated with androgen deprivation: A randomized study. Can Urol Assoc J.

[59] Martinsen S, Flodin P, Berrebi J, Löfgren M, Bileviciute-Ljungar I, Ingvar M, Fransson P, Kosek E (2014). Fibromyalgia Patients Had Normal Distraction Related Pain Inhibition but Cognitive Impairment Reflected in Caudate Nucleus and Hippocampus during the Stroop Color Word Test. PLoS ONE.

[60] Flodin P, Martinsen S, Lofgren M, Bileviciute-Ljungar I, Kosek E, Fransson P (2014). Fibromyalgia is associated with decreased connectivity between pain- and sensorimotor brain areas. Brain Connect.

[61] Emad Y, Ragab Y, Zeinhom F, El-Khouly G, Abou-Zeid A, Rasker JJ (2008). Hippocampus dysfunction may explain symptoms of fibromyalgia syndrome. A study with single-voxel magnetic resonance spectroscopy. J Rheumatol.

[62] Wood PB, Ledbetter CR, Glabus MF, Broadwell LK, Patterson JC (2009). Hippocampal metabolite abnormalities in fibromyalgia: correlation with clinical features. Clin J Pain.

[63] Jones SL, Gebhart GF (1986). Quantitative characterization of ceruleospinal inhibition of nociceptive transmission in the rat. J Neurophysiol.

[64] Al Amin HA, Atweh SF, Jabbur SJ, Saade NE (2004). Effects of ventral hippocampal lesion on thermal and mechanical nociception in neonates and adult rats. Eur J Neurosci.

[65] Maletic V, Robinson M, Oakes T, Iyengar S, Ball SG, Russell J (2007). Neurobiology of depression: an integrated view of key findings. Int J Clin Pract.

[66] Lathe R (2001). Hormones and the hippocampus. J Endocrinol.

[67] McCrae CS, O’Shea AM, Boissoneault J, Vatthauer KE, Robinson ME, Staud R, Perlstein WM, Craggs JG (2015). Fibromyalgia patients have reduced hippocampal volume compared with healthy controls. J Pain Res.

[68] Lutz J, Jager L, de Quervain D, Krauseneck T, Padberg F, Wichnalek M, Beyer A, Stahl R, Zirngibl B, Morhard D (2008). White and gray matter abnormalities in the brain of patients with fibromyalgia: a diffusion-tensor and volumetric imaging study. Arthritis Rheum.

[69] Mutso AA, Radzicki D, Baliki MN, Huang L, Banisadr G, Centeno MV, Radulovic J, Martina M, Miller RJ, Apkarian AV (2012). Abnormalities in hippocampal functioning with persistent pain. J Neurosci.

[70] Apkarian AV, Mutso AA, Centeno MV, Kan L, Wu M, Levinstein M, Banisadr G, Gobeske KT, Miller RJ, Radulovic J (2016). Role of adult hippocampal neurogenesis in persistent pain. Pain.

[71] Erickson KI, Voss MW, Prakash RS, Basak C, Szabo A, Chaddock L, Kim JS, Heo S, Alves H, White SM (2011). Exercise training increases size of hippocampus and improves memory. Proc Natl Acad Sci U S A.

[72] Mueller K, Möller HE, Horstmann A, Busse F, Lepsien J, Blüher M, Stumvoll M, Villringer A, Pleger B (2015). Physical exercise in overweight to obese individuals induces metabolic- and neurotrophic-related structural brain plasticity. Front Hum Neurosci.

[73] Flodin P, Martinsen S, Mannerkorpi K, Lofgren M, Bileviciute-Ljungar I, Kosek E, Fransson P (2015). Normalization of aberrant resting state functional connectivity in fibromyalgia patients following a three month physical exercise therapy. NeuroImage Clin.

[74] Smiley-Oyen AL, Lowry KA, Francois SJ, Kohut ML, Ekkekakis P (2008). Exercise, fitness, and neurocognitive function in older adults: the “selective improvement” and “cardiovascular fitness” hypotheses. Ann Behav Med.

[75] Portugal EM, Vasconcelos PG, Souza R, Lattari E, Monteiro-Junior RS, Machado S, Deslandes AC (2015). Aging process, cognitive decline and Alzheimer’s disease: can strength training modulate these responses?. CNS Neurol Disord Drug Targets.

[76] Floel A, Ruscheweyh R, Kruger K, Willemer C, Winter B, Volker K, Lohmann H, Zitzmann M, Mooren F, Breitenstein C (2010). Physical activity and memory functions: are neurotrophins and cerebral gray matter volume the missing link?. NeuroImage.

[77] Llorens-Martin M, Torres-Aleman I, Trejo JL (2009). Mechanisms mediating brain plasticity: IGF1 and adult hippocampal neurogenesis. Neuroscientist.

[78] Maass A, Duzel S, Brigadski T, Goerke M, Becke A, Sobieray U, Neumann K, Lovden M, Lindenberger U, Backman L (2016). Relationships of peripheral IGF-1, VEGF and BDNF levels to exercise-related changes in memory, hippocampal perfusion and volumes in older adults. NeuroImage..

[79] Trejo JL, Llorens-Martin MV, Torres-Aleman I (2008). The effects of exercise on spatial learning and anxiety-like behavior are mediated by an IGF-I-dependent mechanism related to hippocampal neurogenesis. Mol Cell Neurosci.

[80] Martin SS, Qasim A, Reilly MP (2008). Leptin resistance - A possible interface of inflammation and metabolism in obesity-related cardiovascular disease. J Am Coll Cardiol.

[81] Brandt C, Jakobsen AH, Adser H, Olesen J, Iversen N, Kristensen JM, Hojman P, Wojtaszewski JFP, Hidalgo J, Pilegaard H (2012). IL-6 regulates exercise and training-induced adaptations in subcutaneous adipose tissue in mice. Acta Physiol.

[82] Ropelle ER, Fernandes MFA, Flores MBS, Ueno M, Rocco S, Marin R, Cintra DE, Velloso LA, Franchini KG, Saad MJA (2008). Central Exercise Action Increases the AMPK and mTOR Response to Leptin. PLoS ONE.

[83] Prestes J, Shiguemoto G, Botero JP, Frollini A, Dias R, Leite R, Pereira G, Magosso R, Baldissera V, Cavaglieri C (2009). Effects of resistance training on resistin, leptin, cytokines, and muscle force in elderly post-menopausal women. J Sports Sci.

[84] Fatouros IG, Tournis S, Leontsini D, Jamurtas AZ, Sxina M, Thomakos P, Manousaki M, Douroudos I, Taxildaris K, Mitrakou A (2005). Leptin and adiponectin responses in overweight inactive elderly following resistance training and detraining are intensity related. J Clin Endocrinol Metab.

[85] Myers MG, Leibel RL, Seeley RJ, Schwartz MW (2010). Obesity and leptin resistance: distinguishing cause from effect. Trends Endocrinol Metab.

[86] Kanaley JA, Fenicchia LM, Miller CS, Ploutz-Synder LL, Weinstock RS, Carhart R, Azevedo JL (2001). Resting leptin responses to acute and chronic resistance training in type 2 diabetic men and women. Int J Obes Relat Metab Disord.

